# Genome-Wide Characterization of the *SnRK* Gene Family in *Taxus* and Homologous Validation of *TaSnRK1.2* as a Central Regulator in Stress-Responsive Transcriptional Networks

**DOI:** 10.3390/plants14152410

**Published:** 2025-08-04

**Authors:** Pengjun Lu, Jianqiu Ji, Fangjuan Fan, Tao Liu, Zhenting Shi, Wentao Li, Chongbo Sun

**Affiliations:** 1 Innovation Center of Chinese Medicinal Crops, Zhejiang Academy of Agricultural Sciences, Hangzhou 310000, China; 15851256692@163.com (J.J.); shizhenting@zaas.ac.cn (Z.S.); wtl9004@163.com (W.L.); 2 Institute of Horticulture, Zhejiang Academy of Agricultural Sciences, Hangzhou 310000, China; 3 International Joint Laboratory for Agricultural Plant Metrology and Equipment Innovation, College of Life Sciences, China Jiliang University, Hangzhou 310000, China; 4 Department of Horticultural Technology, School of Forestry Science and Technology, Lishui Vocational and Technical College, Lishui 323000, China; cherish_nu@163.com; 5 Hangzhou Raw Seed Growing Farm, Hangzhou 310000, China; 21216055@zju.edu.cn

**Keywords:** gene family, protoplast transformation, *SnRK*, *Taxus*, transcriptome

## Abstract

SnRK kinases, central regulators of plant stress response, remain uncharacterized in *Taxus*—an ancient gymnosperm valued for paclitaxel production. This study aimed to identify the *Taxus SnRK* family and elucidate its functional roles. Specifically, we identified *SnRK* genes through genomic analysis and assessed tissue-specific expression via transcriptomics, while regulatory networks were deciphered using WGCNA. To overcome experimental constraints, a PEG-mediated protoplast transient expression system was developed using calli, followed by dual-luciferase assays. Consequently, 19 *SnRK* genes (2 *SnRK1*, 4 *SnRK2*, 13 *SnRK3*) were identified, with tissue-specific expression revealing *TaSnRK1.2* upregulation under methyl jasmonate (MeJA) and in stress-resilient tissues (bark/root). Subsequently, WGCNA uncovered a bark/root-specific module containing *TaSnRK1.2* with predicted TF interactions (*TaGRAS*/*TaERF*). Critically, homologous dual-luciferase assays demonstrated *TaSnRK1.2* activates *TaGRAS* and *TaERF* promoters (4.34-fold and 3.11-fold induction, respectively). This study establishes the *Taxus SnRK* family and identifies *TaSnRK1.2* as a hub integrating stress signals (e.g., MeJA) to modulate downstream TF networks, while the novel protoplast system enables future functional studies in this medicinal plant.

## 1. Introduction

Yew (*Taxus spp.*) belongs to the Taxaceae family, which is a group of gymnospermous evergreen shrubs. The toxicity of yew is attributed to taxine alkaloids, specifically Taxol, which is most abundant in the bark but absent in the mature aril [1,2]. The non-toxic aril is gaining recognition as a medicinal food due to its delicious taste and high levels of antioxidants [3,4]. *Taxus* is widely distributed across the Earth and is known as a longevity tree, with a lifespan of thousands of years. Symbolic of eternal life, *Taxus* has the ability to withstand adversity and adapt to plant stress [5,6], making it an excellent model for studying stress adaptation mechanisms. Central to plant stress response are signaling pathways involving kinase cascades. Among these, the *Sucrose non-fermenting related kinases* (*SnRK*) gene family has been extensively studied as a key player in plant stress signaling pathways belonging to the CDPK (calcium-dependent protein kinase)-SnRK superfamily [7]. This superfamily includes seven types of serine–threonine protein kinases, many of which are unique to plants: calcium-dependent protein kinase (CDPKs), CDPK-related kinases (CRKs), phosphoenolpyruvate carboxylase kinases (PPCKs), PEP carboxylase kinase-related kinases (PEPRKs), calmodulin-dependent protein kinases (CaMKs), calcium and calmodulin-dependent protein kinases (CCaMKs), and SnRKs [7]. Within this superfamily, SnRKs play a particularly crucial role in stress signaling. In Arabidopsis, 34 CDPKs, 8 CRKs, 2 PPCKs, 2 PEPRKs, and 38 SnRKs have been identified through genome-wide studies [7]. SnRKs play a central role in phosphorylation cascades in plant stress signaling pathways, such as ABA signaling [7,8]. The SnRK family can be divided into three groups in plants: SnRK1 (energy/signaling sensor), SnRK2 (ABA signaling/osmotic stress), and SnRK3 (Ca^2+^ signaling). SnRK1 is believed to be the ancestral form of plant-specific SnRKs, as seen in red algae (which only contains SnRK1) [8,9]. In Arabidopsis, 3 AtSnRK1, 10 AtSnRK2, and 25 AtSnRK3 have been characterized [7].

Compared to angiosperm species, gymnosperm species have not been extensively studied in terms of *SnRK* genes, with the exception of one study on *Pinus pinaster* SnRKs [10]. In particular, the *SnRK* family genes in *Taxus* have not been studied yet. The phytohormone jasmonate (JA) and its bioactive derivative methyl jasmonate (MeJA) are key signaling molecules involved in plant stress responses and defense. Jasmonate (JA) is a plant hormone involved in stress signaling. Its methyl ester, methyl jasmonate (MeJA), is naturally occurring in plants and is more stable for external application compared to JA [11,12,13,14]. There are also complex interactions between plant hormones and plant–microbe interactions in stress signaling [15,16,17,18]. In *Taxus*, external application of MeJA has been widely studied for stress and Taxol production, with a focus on transcriptional regulation by *MYC*, *ERF*, and other transcription factors [19,20,21,22]. However, there has been no research on post-transcriptional regulation in *Taxus*, including potential regulation by kinases like SnRKs. It is urgent to clarify the relationship between MeJA and *SnRKs* for *Taxus* research and stress-tolerant breeding. Weighted Gene Co-expression Network Analysis (WGCNA) is a method for analyzing correlation matrices of gene expression profiles to identify candidate biomarkers and traits using eigengene network methodology [23]. Correlation networks can be used to find clusters (modules) of interconnected nodes, where genes within each module share a similar expression pattern and have a high correlation coefficient with each other. Genes with higher network connectivity (degree) may have potential regulatory functions. Firefly luciferase (FLUC) is a monomeric protein that does not require post-translational processing to attain enzymatic activity [24]. Therefore, it can serve as a genetic reporter immediately after translation. In assays using FLUC, a transient “flash” of light is generated, which rapidly fades after the substrate and enzyme are mixed. *Renilla* luciferase (RLUC), another monomeric protein similar to FLUC, also does not require post-translational modification for its activity. These two LUCs have distinct substrate requirements and emit light at different wavelengths, making them useful genetic reporters for biological studies [24].

With *Taxus* as the selected genus, two *Taxus* genome datasets (GCA_019776745.2 released in January 2022 and GCA_018340775.1 released in May 2021) are available in the NCBI database (https://www.ncbi.nlm.nih.gov/datasets/genome/?taxon=25628, accessed on 1 June 2025). We selected the recently released *Taxus chinensis* genome (GCA_019776745.2) for *SnRK* family identification. Additionally, we conducted RNA-seq to preliminarily identify tissue-specific expression profiles and their expression patterns under MeJA stress signaling. As an example, we studied a highly expressed member, *TaSnRK1.2*, in detail. To identify potential regulatory targets of *TaSnRK1.2*, we employed Weighted Gene Co-expression Network Analysis (WGCNA) to analyze the co-expression of transcription factors potentially regulated by *TaSnRK1.2*. However, a major obstacle in *Taxus* functional gene research is the lack of a homologous functional gene validation system, as a genetic transformation system has not yet been established. To address this issue, we developed a transient transformation method using protoplasts from *Taxus* calli. This method has been proven to be efficient for gene over-expression and is suitable for single- or dual-luciferase assays to verify the regulation of *TaSnRK1.2* and candidate transcription factors. Furthermore, this method has great potential for gene editing and metabolic engineering in *Taxus*, as demonstrated by successful over-expression of Cas9 and gRNA. This study aims to (1) identify and characterize the *SnRK* gene family in *Taxus*; (2) develop an efficient transient expression platform overcoming *Taxus*’ genetic transformation barriers; and (3) RNA-seq-based screen and functionally validate central regulators in stress-responsive transcriptional networks involving *SnRK* genes.

## 2. Results

### 2.1. Identification and Classification of SnRK Gene Family in Taxus

**Figure 1:**
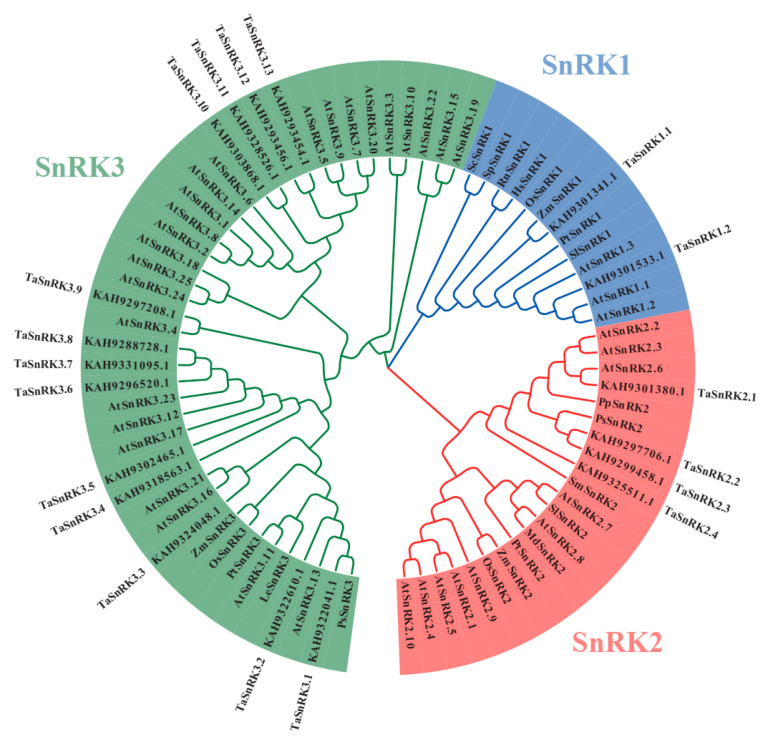
Phylogenetic tree of the SnRK family in *Taxus*, constructed by protein sequences using the neighbor-joining (NJ) method in MEGA 7 with 1000 bootstrap replicates for branch support. SnRKs from Arabidopsis and other species were included to better identify, classify, and name *TaSnRK* family members. Species abbreviations: Cr, *Chlamydomonas reinhardtii*; Hs, *Homo sapiens*; Md, *Malus x domestica*; Os, *Oryza sativa*; Pp, *Physcomitrella patens*; Ps, *Picea sitchensis*; Pt, *Populus trichocarpa*; Rn, *Rattus norvegicus*; Sc, *Saccharomyces cerevisiae*; Sp, *Schizosaccharomyces pombe*; Sm, *Selaginella moellendorffii*; Sl, *Solanum lycopersicum*; Ta, *Taxus*; Zm, *Zea mays*.

The output sequence ID filtered by local hmmsearch was named “set HMMER,” while the output sequence ID filtered by local BLASTP was named “set BLASTP.” The candidate *Taxus* SnRKs ID is the intersection of “set HMMER” and “set BLASTP.” The Pfam database identifies PF00069 as a protein kinase domain, indicating that SnRKs share similarities with other members of the CDPK-SnRK superfamily, such as CDPKs, CRKs, PPCKs, and PEPRKs. To exclude non-SnRKs with high similarity to SnRKs, we constructed a phylogenetic tree that included candidate *Taxus* SnRKs, AtSnRKs, AtCDPKs (CPKs), AtCRKs, AtPPCKs, and AtPEPRKs (Supplementary Figure S1), as well as SnRKs from multiple species (Supplementary Figure S2; Supplementary Table S1). As expected, the phylogenetic tree showed that many CDPKs (CPKs), CRKs, PPCKs, and PEPRKs are included in the “candidate *Taxus* SnRKs” set (Supplementary Figure S3) and need to be excluded from SnRK characterization. A total of 19 TaSnRKs were identified as SnRKs in *Taxus*, with 2 TaSnRK1, 4 TaSnRK2, and 13 TaSnRK3 separated according to their cluster phylogenetic relationships to published SnRK protein sequences from Arabidopsis and other species (Figure 1). The protein ID and sequences of the final TaSnRKs are listed in Supplementary Figure S4.

### 2.2. The Expression Pattern and Profile of SnRK Gene Family by Tissue-Specific and MeJA Addition in the Taxus

**Figure 2:**
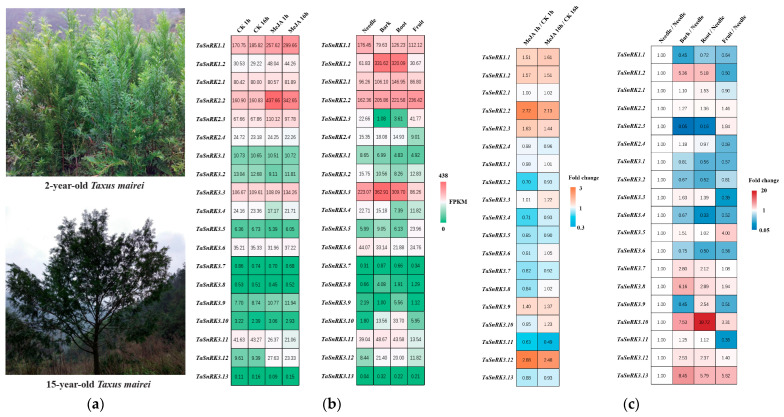
*TaSnRK* gene expression patterns across tissues and MeJA treatment in *Taxus mairei*. (**a**) Morphological characteristics of the *Taxus mairei* plants used in this study; (**b**) heatmap of *TaSnRK* gene expression levels in RNA-seq data across different *Taxus* tissues and in response to methyl jasmonate (MeJA) treatment; (**c**) heatmap of *TaSnRK* gene expression changes (fold change) across *Taxus* tissues and under methyl jasmonate (MeJA) treatment (RNA-seq data).

The mature *Taxus* tree (Figure 2a) is tall with a huge tree crown. In order to easily study it, we selected relatively small 2-year-old *Taxus mairei* trees (Figure 2a) and induced stress using MeJA. We also sampled needles, barks, roots, and green immature fruits (with arils and seeds) from 15-year-old *Taxus mairei* trees to study the expression pattern of the *TaSnRK* family. The RNA-seq results (with three biological replicates) showed that most *TaSnRK*s displayed relatively high expression levels (FPKM > 1) across all tissues and organs, except for two members (*TaSnRK3.7* and *TaSnRK3.13*) with very low expression levels (FPKM < 1), even when treated with MeJA. *TaSnRK1.1*, *TaSnRK1.2*, *TaSnRK2.2*, and *TaSnRK3.12* were upregulated by MeJA with an increase in expression level of more than 1.5 times (Figure 2b). *TaSnRK2.3* and *TaSnRK3.9* were also upregulated by MeJA, while only *TaSnRK3.11* showed a slight decrease in expression (Figure 2b,c). The other *TaSnRK*s did not show a significant response to MeJA. As a long-lived tree with a lifespan of thousands of years, the bark and roots of *Taxus* endure more adversity and coercion. *TaSnRK1.2*, *TaSnRK3.8*, *TaSnRK3.10*, and *TaSnRK3.13* showed a higher expression priority in the bark and roots compared to the needles (fold change > 3). Among them, *TaSnRK1.2* exhibited a high expression abundance in various tissues and organs (FPKM > 30) (Figure 2b). In summary, *TaSnRK1.2*, with its specific expression in the bark and roots, high expression abundance throughout the entire plant, and induction by MeJA, is the best candidate for involvement in stress response.

### 2.3. Weighted Gene Co-Expression Network Analysis (WGCNA) and Screen Out TaSnRK1.2-Related Transcription Factor with Regulatory Relationships by Network Analysis

**Figure 3:**
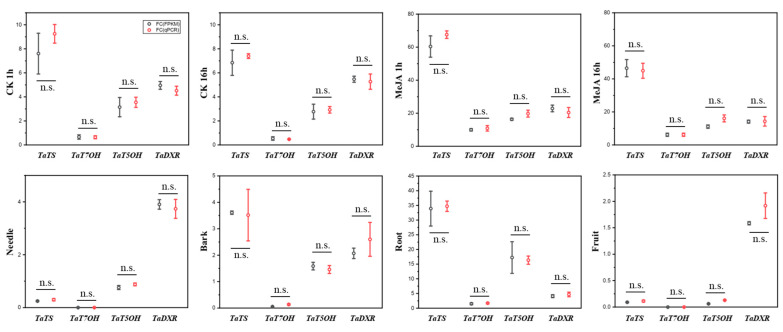
Validation of RNA-seq data by qPCR using randomly selected genes; the dots indicate the average FC (fold change) value (3 biological replicates) of target gene expression level/reference gene expression level. Error bars represent standard errors of three biological replicates. Statistical significance was determined by two-tailed Student’s *t*-test (*p* < 0.05; n.s. = not significant, *p* ≥ 0.05).

Additionally, we conducted qPCR experiments (Supplementary Table S2) to validate the accuracy of our RNA-seq results, as previous studies have shown [25,26,27]. The results confirmed the consistency between qPCR and RNA-seq (Figure 3). Thus, the RNA-seq-based screening for stress-responsive transcriptional networks and hub genes reflects the objective and true gene expression situation.

**Figure 4:**
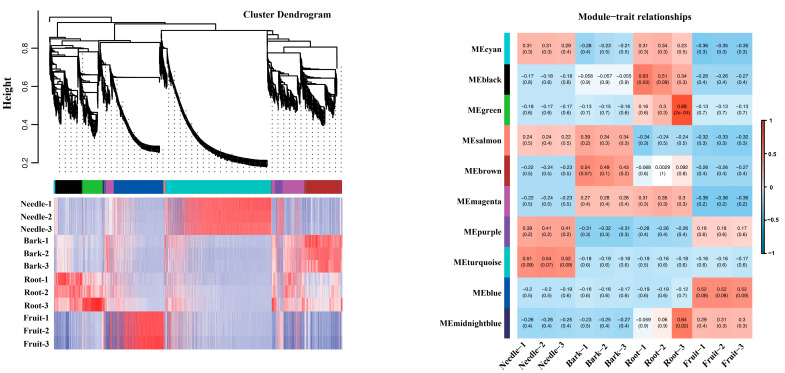
Hierarchical clustering dendrogram for co-expression module construction and heatmap of module–trait correlations in WGCNA analysis. Hierarchical clustering dendrogram of genes based on topological overlap dissimilarity (1-TOM). Branches are color-coded to represent distinct co-expression modules identified by the dynamic tree cut algorithm. The height scale (*y*-axis) indicates the dissimilarity level at which clusters are merged. Heatmap of module–trait correlations. Each row corresponds to a co-expression module (labeled by module color), and each column represents a phenotypic trait. Color intensity reflects the Pearson correlation coefficient (r) between the module eigengene and the trait, as indicated by the color scale bar (right). Red hues denote positive correlations (r > 0), blue hues denote negative correlations (r < 0), and white indicates no correlation (r ≈ 0).

We then used WGCNA to identify bark/root-specific genes and constructed a network heatmap plot with corresponding hierarchical clustering dendrograms (Figure 4). From this, we selected several representative modules for further analysis of the module–trait relationship, specifically looking at tissue-specific expression patterns (Figure 4). One module in particular, the magenta module, showed a high correlation (indicated by the red color in the heatmap) in both bark- and root-specific expression (Figure 4).

**Figure 5:**
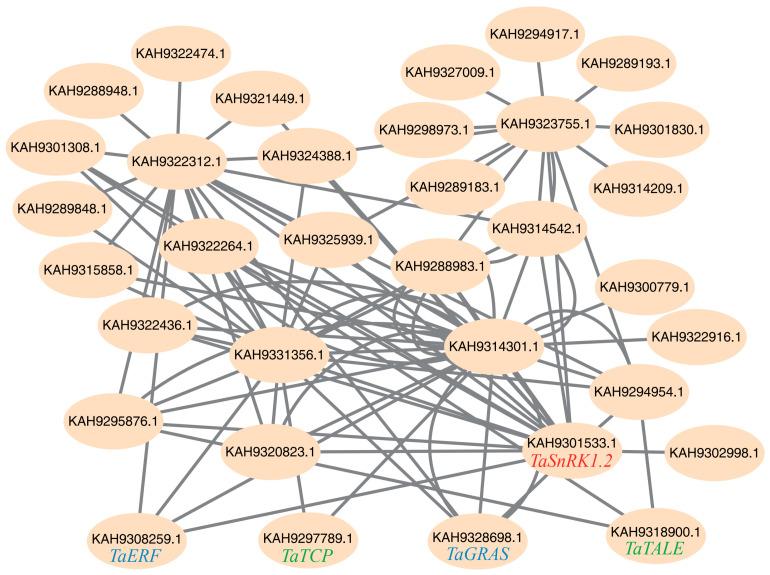
Co-expressed regulatory networks in the magenta module in WGCNA analysis. The network depicts hub genes and their interacting partners with edges representing significant co-expression relationships. Top 90 genes with connectivity values from high to low are extracted for co-expressed regulatory network construction.

In WGCNA based on scale-free network topology, genes with high intramodular connectivity exhibit extensive co-expression relationships, positioning them at topologically central layers of the regulatory hierarchy. Such highly connected genes are thus defined as hub genes, which typically perform critical biological functions. Within this module, we found *TaSnRK1.2*, which aligned with our expectations. We then sorted the top 90 genes (including *TaSnRK1.2*) in the magenta module based on their connectivity values in ascending order and visualized them using Cytoscape v3.10.3 for co-expression network calculation and analysis (Figure 5). Four of these genes were identified as transcription factors by the Plant Transcription Factor Database [28]: *TaERF*, *TaGRAS*, *TaTCP*, and *TaTALE*.

**Figure 6:**
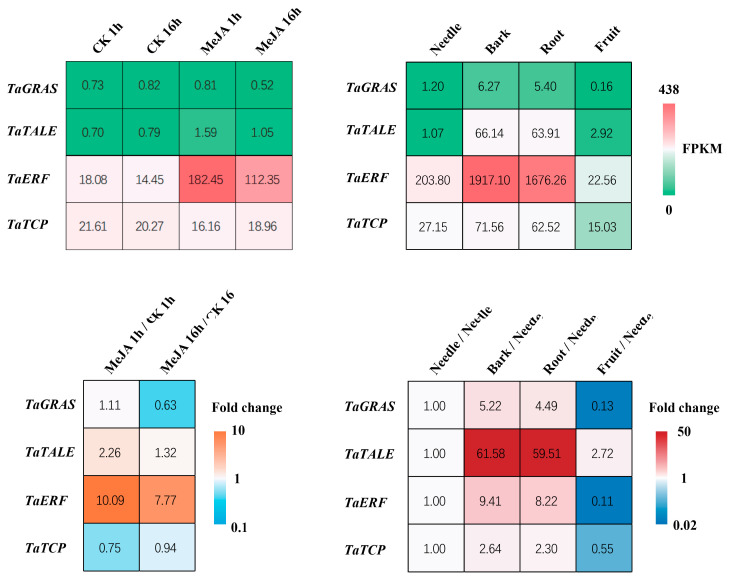
Heatmap of the four newly identified transcription factors’ gene expression levels and fold changes across different *Taxus* tissues and in response to methyl jasmonate (MeJA) treatment.

All four of these transcription factors showed tissue-specific expression patterns that aligned with the characteristics of the magenta module (Figure 6). We also found that MeJA spraying greatly upregulated the transcription levels of *TaERF*, moderately upregulated the transcription levels of *TaTALE*, and had little to no impact on the transcription levels of *TaGRAS* and *TaTCP* (Figure 6). In our network analysis, we predicted that *TaERF* and *TaGRAS* have a regulatory relationship with *TaSnRK1.2*. As transcription factors often regulate multiple downstream target genes and have high connectivity values in scale-free topology networks, their regulation serves as an indicator of the capacity to modulate the entire stress response network. Therefore, we conducted experiments to determine whether *SnRK* regulates these two transcription factors, *TaERF* and *TaGRAS*.

### 2.4. The Development of Protoplast Transformation in Taxus

**Figure 7:**
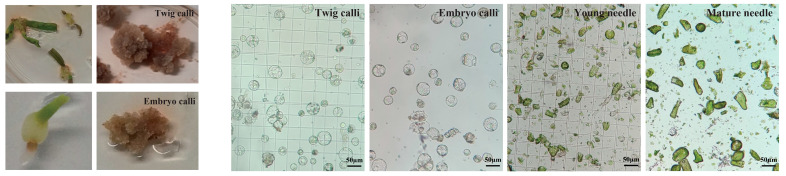
Protoplast morphology under microscopy following enzymatic isolation from four *Taxus* tissue groups, including calli induced from embryonic tissues and twigs.

**Figure 8:**
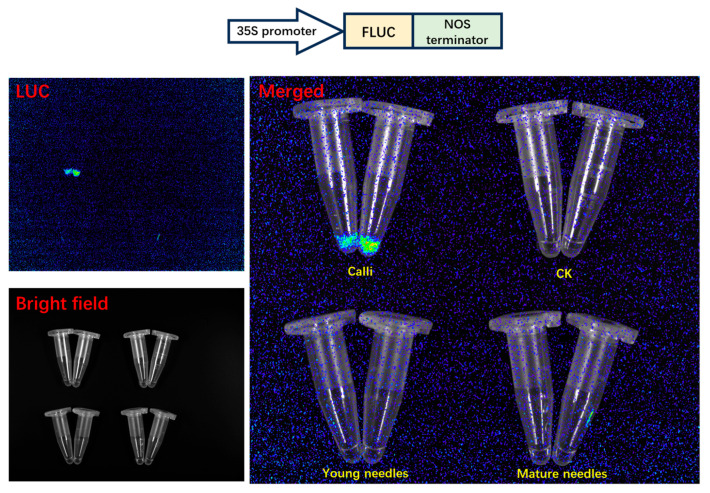
Single-luciferase reporter assay used for evaluating transient expression efficiency in transiently transformed protoplasts.

The prolonged growth cycle and the tendency towards browning pose significant obstacles to achieving stable genetic transformation in *Taxus*. To address this, we have developed a protoplast transient expression system for *Taxus* to conduct homologous validation of the regulatory relationship between *TaSnRK1.2* and two transcription factors (*TaERF* and *TaGRAS*) investigated in this study. To thoroughly evaluate the physiological state and viability of protoplasts derived from various tissue and organ sources, and to optimize the efficiency of transient transformation experiments, we meticulously selected four distinct experimental groups. These groups included calli that had been initiated from twigs (Figure 7), calli derived from embryonic tissues (Figure 7), fresh young needles, and fully developed mature needles. For each of these groups, we carried out enzymatic digestion to break down the plant cell walls, thereby releasing intact and functional protoplasts for subsequent experimental analyses. Filtered by a 100 μm nylon mesh, protoplasts harvested from both calli showed good morphology and high yield (Figure 7). Microscopic examination of the young needles group revealed a large number of debris and contaminants, along with a relatively low yield of protoplasts (Figure 7). In contrast, the enzymatic digestion of mature needles yielded almost no protoplasts (Figure 7). PEG–calcium-mediated Pro35S-LUC plasmid was transiently transformed into protoplasts from calli (mixing two types of calli in equal amounts), young needles, and mature needles. The results clearly showed that the calli group achieved a highly satisfactory transgenic outcome (Figure 8). The luminescence intensity was almost undetectable in the other two groups (Figure 8), which was consistent with the expectations based on the microscopic examination results of protoplasts.

### 2.5. TaSnRK1.2 Upregulates the Expression of Both TaGRAS and TaERF in Taxus

**Figure 9:**
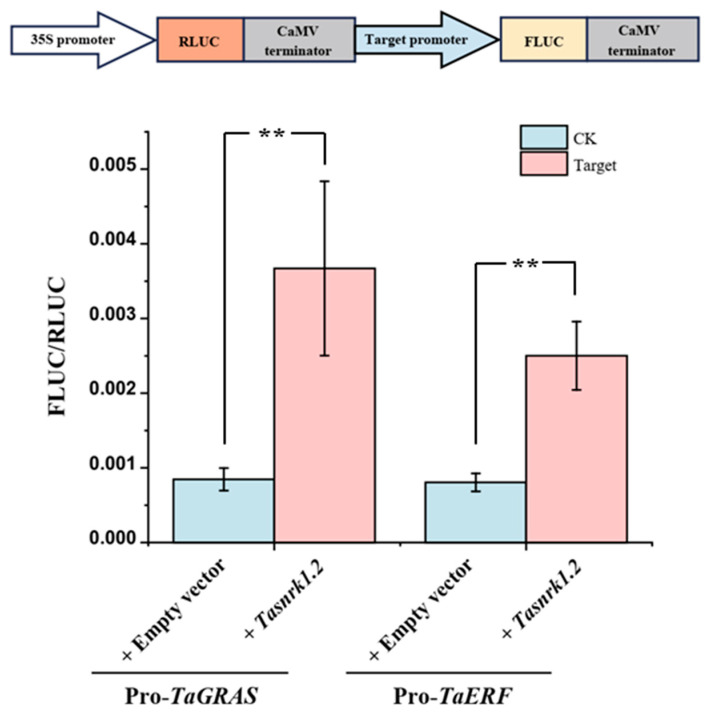
Dual-luciferase reporter assay used to verify the regulation of *TaSnRK1.2* and candidate transcription factors. The error bars represent the standard error of biological replicates. The independent sample two-tailed Student’s *t*-test was used for statistical analysis (** *p*  <  0.01).

Based on the efficient protoplast transient expression system established in this study, the regulatory relationship between *TaSnRK1.2* and two transcription factors (*TaGRAS* and *TaERF*) was further studied using a dual-luciferase assay. The 2000 bp sequence upstream of the start codon (ATG) was cloned and used to construct a dual-luciferase reporter system plasmid (Supplementary Figure S5). The plasmid contained the firefly luciferase (FLUC) gene controlled by the promoter of interest and the *Renilla* luciferase (RLUC) gene expressed under a 35S promoter (Figure 9). The results showed that, compared to the control group, *TaSnRK1.2* + Pro*TaGRAS* showed a 4.34-fold increase in reporter signal and *TaSnRK1.2* + Pro*TaERF* showed a 3.11-fold increase, indicating that *TaSnRK1.2* activates both *TaGRAS* and *TaERF* genes (Figure 9).

## 3. Discussion

This study makes three significant contributions: (1) The first comprehensive identification of the *SnRK* gene family in *Taxus*, revealing evolutionary conservation with angiosperms through reduced gene numbers (19 *TaSnRK*s compared to 38 in Arabidopsis [7]) and similarities to *Pinus pinaster* [10]. (2) Development of a high-efficiency protoplast system that overcomes *Taxus*’ recalcitrance to genetic studies, enabling homologous validation of regulatory networks. (3) Characterization of *TaSnRK1.2* as a central stress response hub. *TaSnRK1.2* exhibits high expression across all examined tissues, is significantly upregulated in response to the stress hormone methyl jasmonate (MeJA), and shows preferential expression in bark and root tissues (both critical for enduring environmental adversity and taxane biosynthesis) (Figure 2b,c), integrating developmental and stress responses in *Taxus*. This aligns with previous studies in model plants, where *SnRK1* subfamily members often act as central regulators of energy sensing and stress adaptation [7,29]. To further understand the regulatory network governed by *TaSnRK1.2*, we employed experimental study. WGCNA identified *TaERF* and *TaGRAS* as *TaSnRK1.2*-associated transcription factors (TFs) (Figure 5). *ERF* transcription factors are well-established downstream components of JA signaling and stress responses [16,18,30], while *GRAS* factors often regulate development and stress adaptation [31,32]. The observed upregulation of *TaGRAS* and *TaERF* by *TaSnRK1.2* suggests a hierarchical regulatory network where SnRK kinases act as upstream modulators of transcription factor activity, thereby integrating stress signals with downstream physiological responses. Dual-luciferase assays confirmed that *TaSnRK1.2* activates *TaERF* and *TaGRAS* promoters (Figure 9). *TaERF* is directly induced by MeJA (Figure 6), aligning with its role in JA signaling [16,30]. *Taxus* employs *TaSnRK1.2* as the primary JA-responsive kinase, indicating gymnosperm-specific adaptation to stress resilience.

The methodological advancement (development of a PEG–calcium-mediated protoplast transient expression system using *Taxus* calli) overcomes the formidable barriers to stable transformation in *Taxus*, such as slow growth, recalcitrance to tissue culture, and browning. It has proven to be highly efficient for plasmid delivery, as evidenced by robust luciferase reporter expression (Figure 8). Crucially, this system enables the homologous validation of predicted regulatory interactions within *Taxus* cells. With this system, we can not only validate TF–promoter interactions within homologous systems like this study, but also address a critical bottleneck in *Taxus* functional genomics. Its applicability for dual-luciferase assays, as demonstrated here, opens avenues for rapidly screening promoter activities, validating TF–target gene interactions, and assessing protein–protein interactions (e.g., using bimolecular fluorescence complementation assays, BiFC). Furthermore, the system holds immense promise for future metabolic engineering efforts aimed at enhancing Taxol production. Transient over-expression of key biosynthetic genes or transcription factors, or crucially, the implementation of CRISPR-Cas9 genome editing (by co-delivering Cas9 and sgRNA constructs), becomes feasible with this platform, bypassing the need for stable transformation. This finding sheds light on post-translational modification (phosphorylation) and transcriptional control in *Taxus* stress biology.

Despite significant advances in our understanding of *TaSnRK1.2* and its role in regulating *TaGRAS* and *TaERF*, there are still limitations that need to be addressed. While our results provide insight into these regulatory mechanisms, further experimental work is necessary to systematically verify them. As a phosphorylation kinase, SnRK has the ability to modulate signal networks through complex and diverse mechanisms [7,8,29]. Specifically, it can either phosphorylate certain transcription factors to enhance their binding to the promoters of *TaGRAS* and *TaERF*, thereby promoting their transcription, or phosphorylate inhibitory transcription factors to facilitate their degradation, ultimately leading to the activation of target genes. While this study has not yet conducted detailed experimental validation, the *Taxus* protoplast system developed here provides a strong technical foundation for future investigations. This platform allows for comprehensive mechanistic studies through techniques such as homologous co-immunoprecipitation (Co-IP), bimolecular fluorescence complementation (BiFC) assays, yeast hybrid (Y1H or Y2H), DNA pull-down, and electrophoretic mobility shift assays (EMSA), which can provide detailed insights into the signal transduction pathways and transcriptional regulatory networks involved. Additionally, further research on other members of the SnRK subfamily, particularly those with tissue-specific expression profiles, is crucial for a more complete understanding of their roles in *Taxus*. For example, while *SnRK2* in angiosperms participates in ABA signaling alongside protein phosphatase 2C (PP2C) [8], how this mechanism may differ in the gymnosperm *Taxus* under water deficit remains unclear. This has broad implications for addressing drought and water scarcity, as well as shedding light on the evolutionary significance of the transition from aquatic to terrestrial life.

## 4. Materials and Methods

### 4.1. Gene Identification and Phylogenetic Analysis

Pfam Protein kinase domain (PF00069), which is representative of SnRK, was downloaded from the Inter Pro Scan database (https://www.ebi.ac.uk/interpro/entry/pfam/PF00069/, accessed on 1 June 2025). The software HMMER 3.0 [33] was used to perform local hmmsearch v3.0 on annotated protein sequences from *Taxus* (https://www.ncbi.nlm.nih.gov/datasets/genome/GCA_019776745.2/, accessed on 1 June 2025) with a cut-off E-value of <1 × 10^−20^ for both full-length and best domain. Protein sequences of AtSnRKs, AtCDPKs (CPKs), AtCRKs, AtPPCKs, and AtPEPRKs from Arabidopsis [7] were downloaded from NCBI (https://www.ncbi.nlm.nih.gov/, accessed on 1 June 2025) (Supplementary Figure S1). Local BLASTP was then performed using the protein AtSnRKs sequences as query and annotated protein sequences from *Taxus* as the database, with default parameters and was further filtered with a threshold of identity > 45. The candidate sequence was further confirmed in the Conserved Domain Database (CDD) (https://www.ncbi.nlm.nih.gov/Structure/cdd/wrpsb.cgi, accessed on 1 June 2025) to remove the false-positive sequences.

The protein sequences of SnRKs from various species were obtained from a previous study [34]. The sequences and corresponding ID information can be found in Supplementary Figure S2 and Supplementary Table S1. Multiple sequence alignments were performed using the CLUSTAL tool in MEGA 7.0 software [35] with default settings. Any sequences with poor alignment were excluded. The phylogenetic tree was constructed using the neighbor-joining method with 1000 bootstrap replicates in MEGA 7.0 software with default settings. The tree displayed only in topology with circle style. The cut-off value for the condensed tree was 50%. To enhance the visual presentation of the tree, Evolview (http://www.evolgenius.info/evolview/, accessed on 1 June 2025) was utilized [36].

### 4.2. Plant Materials and Treatment

All experimental data in this study are performed with three biological replicates ensuring data robustness. The 2-year-old *Taxus mairei* was used for external application of spraying MeJA (100 μM). The branches (stems and needles) were cut off, immediately frozen in liquid nitrogen, and stored at −70 °C for further analysis. The 15-year-old *Taxus mairei* was used for sampling of different organs and tissues and stored as described above. Germinated seeds of *Taxus mairei* were sterilized with 75% ethanol for 5 min and 1% sodium hypochlorite solution for 20 min. The seeds with the seed coat removed were placed on MS solid media [37] for one week to increase seed vigor before being transferred to calli induction media. B5 solid media [38] with kinetin (0.1 mg/L) and gibberellic acid (0.5 mg/L) were used for *Taxus* calli induction, as reported in previous studies [39,40]. Twigs with needles were cut off and soaked in 75% ethanol for 5 min, followed by 1% sodium hypochlorite solution for 20 min for sterilization. The disinfected twig explants were then patted dry on filter paper and placed directly on B5 solid media for calli induction.

### 4.3. RNA Extraction, RNA-Seq Analysis, and Quantitative Real-Time PCR (qPCR) Analysis

Total RNA was extracted using the FastPure Universal Plant Total RNA Isolation Kit (Vazyme, Nanjing, China). The extracted RNA was of high quality, with an RNA integrity value of over seven, and was used for RNA-seq analysis by Lianchuan company (Hangzhou, China) on an Illumina HiSeq PE150 platform (Illumina, San Diego, CA, USA). Real-time quantitative PCR was performed using the Biorad-CFX96 Q-PCR instrument (Bio-Rad, Hercules, CA, USA) with the HiScript II 1st Strand cDNA Synthesis Kit (Vazyme, Nanjing, China) and SYBR Green qPCR Master Mix (MedChemExpress, Monmouth Junction, NJ, USA,). All procedures were followed according to the instruction manual. The PCR program consisted of an initial step of 5 min at 95 °C, followed by 45 cycles of 95 °C for 5 s, 58 °C for 15 s, and 72 °C for 10 s. A melting curve was generated for each sample at the end of each run to assess the purity of the amplified products. The reference gene *TaARP2* (actin-related protein) was used to normalize the mRNA levels for each sample, as per previous research [26]. Other qPCR primers used for the fidelity of RNA-seq were referenced from previous research and verified in the *Taxus* genome sequence. The primers are listed in Supplementary Table S2. *TaTS*, *TaT7OH*, and *TaT5OH* are from [25] and *TaDXR* is from [27].

### 4.4. Weighted Gene Co-Expression Network Analysis (WGCNA) and Establishment of Co-Expression Networks

To identify clusters (modules) of highly correlated genes, we utilized differentially expressed genes and performed WGCNA using the R-package WGCNA [23]. The co-expression adjacency matrix was created by calculating the correlation between each gene, which was then converted into a topological overlap (TO) matrix. The modules were clustered based on TO similarity and a dynamic tree cut was generated to identify similar modules. The hierarchical clustering dendrogram of genes was based on topological overlap dissimilarity (1-TOM). Branches were color-coded to represent distinct co-expression modules identified by the dynamic tree cut algorithm. The height scale (*y*-axis) indicates the dissimilarity level at which clusters are merged. In the heatmap of module–trait correlations, each row corresponds to a co-expression module (labeled by module color), and each column represents a phenotypic trait. Color intensity reflects the Pearson correlation coefficient (r) between the module eigengene and the trait, as indicated by the color scale bar (right). Red hues denote positive correlations (r > 0), blue hues denote negative correlations (r < 0), and white indicates no correlation (r ≈ 0). The connectivity of a gene was determined by its correlation with all other genes in the network. To visualize the co-expression networks, we used the Cytoscape v3.10.3 software [41] to display genes that potentially interact within a module. Transcription factor genes were predicted using the Plant Transcription Factor Database (https://planttfdb.gao-lab.org/, accessed on 1 June 2025) [28].

### 4.5. PEG–Calcium-Mediated Protoplast Transformation in Taxus

*Taxus* protoplast isolation and PEG–calcium transfection of plasmid DNA is based on previous research on Arabidopsis [42] with some modifications in this study. A fresh enzyme solution (20 mM MES, 1.0% *w*/*v* cellulase R10, 0.5% *w*/*v* macerozyme R10, 0.15% *w*/*v* pectolyase Y-23, 0.5 M mannitol, 20 mM KCl, 10 mM CaCl_2_, 0.1% BSA, pH = 5.6) was prepared before transferring *Taxus* calli or needles into it. Young needles with light green color and mature needles with dark green color were cut into 1 mm wide strips using a sharp razor blade. The calli and needle strips were then immersed in the enzyme solution and vacuum infiltrated for 10 min. The mixture was incubated at room temperature for 2 h and the protoplast state was checked under a microscope. Typically, 3–5 h is suitable for protoplast release. The protoplasts were then filtered through a 100 mm nylon mesh and an equal volume of W5 buffer (154 mM NaCl, 125 mM CaCl_2_, 5 mM KCl, pH = 5.6) was added. The protoplasts were allowed to settle at the bottom of the tube by gravity and kept on ice for 30 min. The supernatant was then removed and the protoplasts were washed with W5 buffer again. The mixture was centrifuged at 100 g (approximately 800 rpm) for 2 min and the protoplasts were collected at the bottom. The protoplasts were re-suspended in a small amount of W5 buffer and the total number was calculated using a hemacytometer under the microscope. The protoplasts were then re-suspended in MMG buffer (0.4 M mannitol, 15 mM MgCl_2_, 4 mM MES, pH = 5.6) to a density of approximately 1 × 105 mL^−1^ for DNA–PEG–calcium transfection. The concentration of the purified plasmid solution was adjusted to around 1–2 μg/μL. An amount of 10 μL of the plasmid solution was added to a round-bottomed centrifuge tube, followed by 100 μL of protoplast suspension and 110 μL of PEG–calcium transfection solution (40% *w*/*v* PEG4000, 0.2 M mannitol, 100 mM CaCl_2_). The tube was gently tapped to mix the solutions and incubated for 15 min. An amount of 440 μL of W5 buffer was then added and the tube was gently tapped to stop the transfection process. The mixture was centrifuged at 100 g for 2 min and the supernatant was removed. The protoplasts were then gently washed and re-suspended in 1 mL of WI buffer (4mM MES, 0.5 M mannitol, 20 mM KCl, pH = 5.6) and transferred to a 6-well tissue culture plate coated with 1–5% BSA (bovine serum albumin). The transfected protoplasts were incubated in the dark for 48 h at room temperature.

### 4.6. Protoplast Single- or Dual-Luciferase Assay

Protoplasts were harvested from one well and vigorously vortexed to break them up for the single- or dual-luciferase assay. The Dual-Luciferase Reporter Assay System E1910 kit (Promega, Madison, WI, USA) was used according to the instruction book. LUC imaging was performed using GelView 6000Plus and luminous intensity was calculated using Promega GloMax.

## Data Availability

Data and material of this study are available from the corresponding author upon reasonable request.

## Supplementary Materials

The following supporting information can be downloaded at: https://www.mdpi.com/article/10.3390/plants14152410/s1, Supplementary Table S1: Protein sequences of SnRKs from multiple species to construct phylogenetic tree; Supplementary Table S2: Primers for qPCR; Supplementary Figure S1: Protein sequences of AtSnRKs, AtCDPKs (CPKs), AtCRKs, AtPPCKs, and AtPEPRKs; Supplementary Figure S2: Protein sequences of SnRKs from multiple species; Supplementary Figure S3: Phylogenetic reconstruction of candidate *Taxus* SnRKs, Arabidopsis kinases (SnRKs, CDPKsCPKs, CRKs, PPCKs, PEPRKs), and orthologous SnRKs from diverse species; Supplementary Figure S4: The protein ID and sequences of the final identified TaSnRKs; Supplementary Figure S5: The 2000 bp sequence upstream of the start codon (ATG) was cloned and used to construct a dual-luciferase reporter system plasmid.

## Abbreviations

The following abbreviations are used in this manuscript:

TFs	transcription factors
MeJA	methyl jasmonate
SnRK	Sucrose non-fermenting related kinases
CDPK	calcium-dependent protein kinase
CRK	CDPK-related kinase
PPCK	phosphoenolpyruvate carboxylase kinase
PEPRK	PEP carboxylase kinase-related kinase
CaMK	calmodulin-dependent protein kinase
CCaMK	calcium and calmodulin-dependent protein kinase
WGCNA	Weighted Gene Co-expression Network Analysis
FLUC	firefly luciferase
RLUC	*Renilla* luciferase
Co-IP	co-immunoprecipitation
BiFC	bimolecular fluorescence complementation
EMSA	electrophoretic mobility shift assays
qPCR	quantitative real-time PCR
