# Supplementary material for: Genome-Wide Characterization of the SnRK Gene Family in Taxus and Homologous Validation of TaSnRK1.2 as a Central Regulator in Stress-Responsive Transcriptional Networks
Source: Plants (Basel). 2025 Aug 4;14(15):2410. doi: 10.3390/plants14152410 (PMC12349213; doi:10.3390/plants14152410)
Supplement: Supplementary file 1 [file plants-14-02410-s001.zip › plants-3713812-supplementary/Supplementary Figure S3.Phylogenetic reconstruction of candidate Taxus SnRKs, Arabidopsis kinases (SnRKs, CDPKsCPKs, CRKs, PPCKs, PEPRKs), and orthologous SnRKs from diverse species (2).pdf]

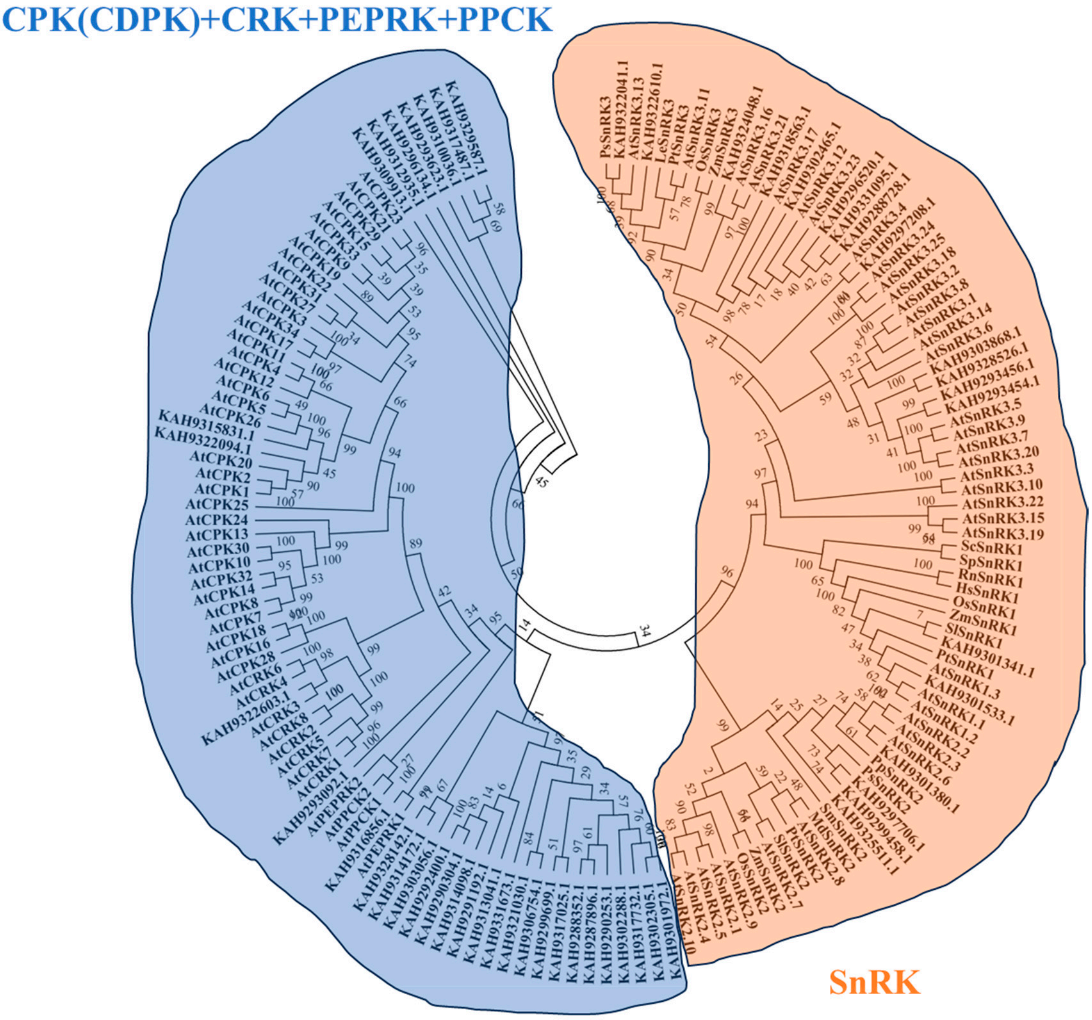

Supplementary Figure S3. Phylogenetic reconstruction of candidate *Taxus* SnRKs, Arabidopsis kinases (SnRKs, CDPKs/CPKs, CRKs, PPCKs, PEPRKs), and orthologous SnRKs from diverse species.
