# Supplementary material for: Genome-Wide Characterization of the SnRK Gene Family in Taxus and Homologous Validation of TaSnRK1.2 as a Central Regulator in Stress-Responsive Transcriptional Networks
Source: Plants (Basel). 2025 Aug 4;14(15):2410. doi: 10.3390/plants14152410 (PMC12349213; doi:10.3390/plants14152410)
Supplement: Supplementary file 1 [file plants-14-02410-s001.zip › plants-3713812-supplementary/Supplementary Table S2. Primers for qPCR.pdf]

Supplementary Table S2. Primers for qPCR.

Gene Name	Protein ID	Primer (5'-3')
TaARP2 (reference gene)	KAH9312167.1	F-AACTACACGCTTCCAGATG<br>R-TCCTCCGCTCAGAACAAT
TaTS	KAH9297158.1	F-TTCGCACGCACGGATACG<br>R-TTCACCACGCTTCTCAATTCG
TaT7OH	KAH9296915.1	F-GGTCCGCCCAAATTGCCAGAA<br>R-CCCTGCAGAGCCCAAAAAACCT
TaT5OH	KAH9297121.1	F-ATGCCTCCTATGACACCACC<br>R-GATTTCTTCGCCCTCCTCTT
TaDXR	KAH9330057.1	F-TGGAGAAACTGAAGGAGGTA<br>R-CTTATTGAACAGGGTGGC
